# Time of day of induction impacts the total duration of labor

**DOI:** 10.1016/j.ajogmf.2026.101898

**Published:** 2026-01-21

**Authors:** Kylie Cataldo, Robert Long, Isoken Olomu, Rene Cortese, Hanne M. Hoffmann

**Affiliations:** Departments of Pediatrics and Obstetrics, Gynecology, and Women’s Health, University of Missouri, Columbia, MO; MU Institute for Data Science and Informatics, University of Missouri, Columbia, MO; Department of Obstetrics and Gynecology, Michigan State University, East Lansing, MI; Division of Neonatology, Department of Pediatrics and Human Development, Michigan State University, East Lansing, MI; Departments of Pediatrics and Obstetrics, Gynecology, and Women’s Health, University of Missouri, Columbia, MO; MU Institute for Data Science and Informatics, University of Missouri, Columbia, MO; Department of Animal Sciences and the Reproductive and Developmental Sciences Program, Michigan State University, East Lansing, MI

**Keywords:** cesarean delivery, circadian rhythm, induction of labor, labor duration, obesity, parity, time-of-day

## Abstract

**BACKGROUND:**

Spontaneous labor onset peaks during the late evening and early morning hours, indicating a circadian influence on parturition. However, the effect of the time of day of labor induction on labor duration and obstetrical outcomes remains unexplored. We hypothesize that the time of day of labor induction affects induction of labor duration and the risk of cesarean delivery.

**OBJECTIVE:**

This study aimed to determine whether the time of day of labor induction impacts induction of labor duration and delivery outcomes in term pregnancies, and whether maternal characteristics such as body mass index and parity modulate this effect.

**STUDY DESIGN:**

This retrospective cohort study analyzed 3363 term pregnant participants who underwent induction at a single US hospital between 2019 and 2022. Time of induction was defined as the time of administration of the first cervical ripening agent or synthetic oxytocin (ie, Pitocin). Induction of labor duration was calculated as the time from induction to delivery. Multivariable analyses, survival models, and circadian rhythm analyses were performed to evaluate associations between time of day of labor induction, induction of labor duration, cesarean delivery, and neonatal outcomes.

**RESULTS:**

Induction of labor duration followed a significant circadian rhythm (*P*<.05, Lomb–Scargle), with a gradual lengthening in duration when induction was initiated later in the day, peaking at 11:00 pm (average duration of 21.0 vs 14.8 hours at 5:00 am; *P*<.01, Kruskal–Wallis test). Participants induced during the early morning had up to 6 hours shorter labor compared with those induced in the late evening (*P*<.01). The optimal time of day for initiating labor induction was influenced by body mass index and parity, with significant differences in delivery probability by time of day of labor induction among nulliparous obese (*P*<.05, 2-way analysis of variance), and parous obese participants (*P*<.05). Time of induction was associated with reduced cesarean delivery rates and did not impact rates of neonatal intensive care unit admission or adverse neonatal outcomes.

**CONCLUSION:**

The time of day when labor induction was initiated influenced induction of labor duration, with the shortest duration observed when induction occurred during early morning hours. No increase in adverse maternal or fetal outcomes was identified after accounting for the time of day of labor induction. The optimal time of day for inducing labor is influenced by body mass index and parity and should be considered when performing this common obstetrical intervention.

## Introduction

Following the publication of the ARRIVE (A Randomized Trial of Induction Versus Expectant Management) trial, labor induction has become an increasingly common obstetrical intervention.^1^ Induction of labor (IOL) is indicated when maternal and fetal outcomes are expected to improve with intervention.^2^ Common indications for labor induction include risk reduction after 39 weeks of gestation,^1^ late-term pregnancy,^3^ premature rupture of membranes,^4^ gestational diabetes,^5^ and preeclampsia.^6^

Synthetic oxytocin (ie, Pitocin), is used to induce labor by stimulating uterine contractions, mimicking the role of oxytocin during spontaneous labor.^7^ It is well established that spontaneous labor and birth peak during late evening and early morning hours,^8,9^ indicating that circadian mechanisms contribute to labor onset. The contribution of the circadian system to labor onset is supported by prior research demonstrating circadian release of oxytocin in both mice and humans.^10,11^ Furthermore, the mouse uterus has circadian rhythms,^12^ with oxytocin receptor knockout mice lacking circadian gating of labor onset.^11^ In addition, we^13–16^ and others^14^ have identified that participants with preterm birth, preeclampsia, and gestational diabetes have deregulated expression of molecular clock genes that drive circadian rhythms on a cellular level, indicating that deregulation of the body’s circadian timekeeping system is associated with poor pregnancy outcomes.

Despite extensive studies demonstrating the relationship between circadian rhythms and labor, the timing of IOL has yet to be considered. Herein, we describe a relationship between the time of day of initiating labor induction (TOI) and labor duration.

## Materials and methods

### Participants

**FIGURE 1:**
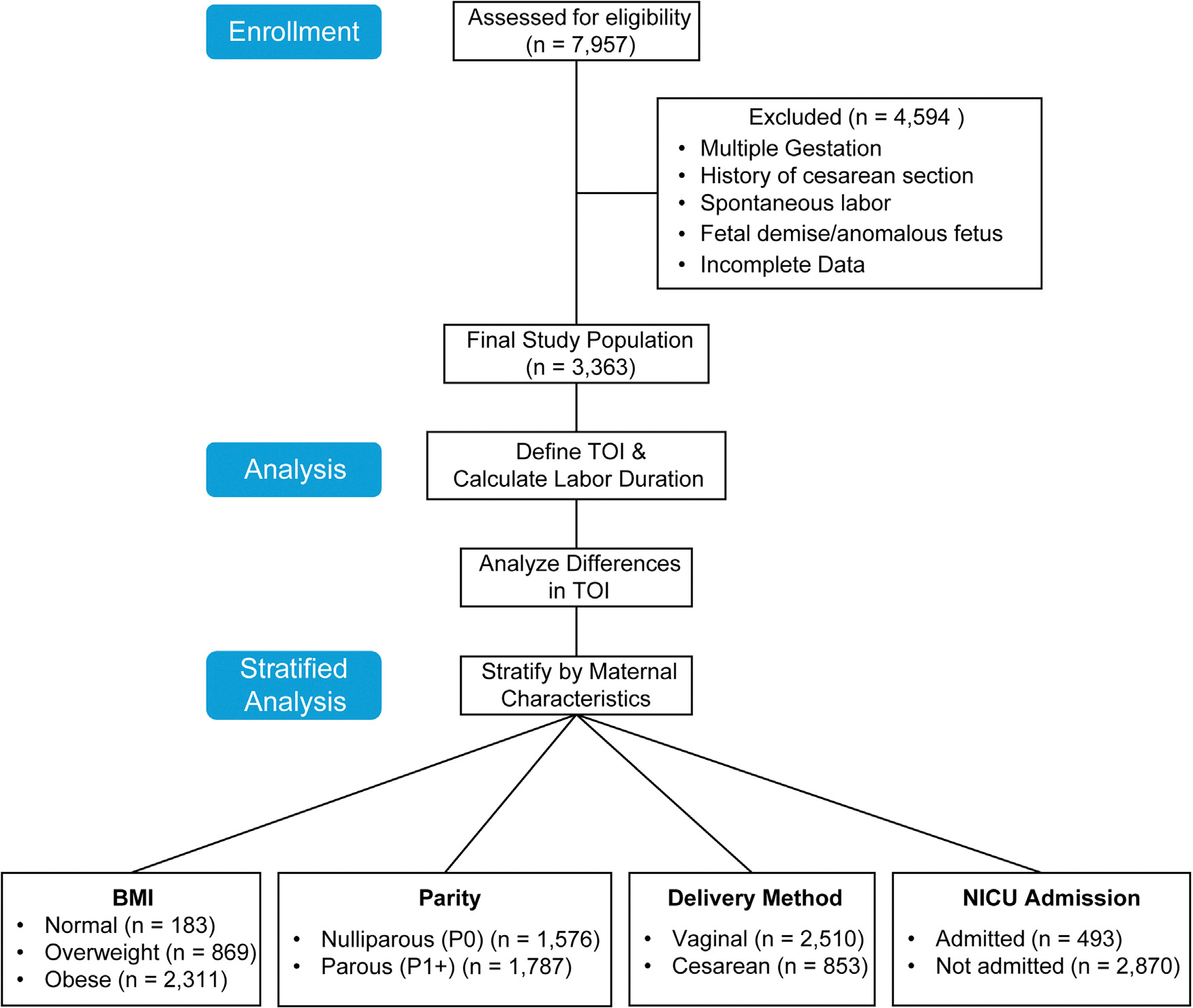
Flowchart showing the participant selection Deidentified data were collected from electronic health records for 7957 deliveries. Participants with incomplete data, history of cesarean delivery, multiple gestation pregnancies, spontaneous labor, or fetal anomalies/demise were excluded from the data set (n=4594), resulting in 3363 deliveries for the final study population. Induction of labor duration was calculated to analyze differences in TOI. A more granular stratified analysis was then performed to analyze differences in TOI across normal weight (n=183), overweight (n=869), and obese (n=2311) BMI groups, nulliparous (P0) (n=1576) and parous (P1+) (n=1787) participants, vaginal (n=2510) and cesarean (n=853) deliveries, and NICU admission (n=493 admitted; n=2870 not admitted).

Deidentified data were collected from electronic health records corresponding to 7957 consecutive deliveries at >37 weeks of gestation at Sparrow Health System, East Lansing, Michigan from February 2019 to March 2022 with approval from the Institutional Review Board of Michigan State University (Study ID: 0007199). Participants with incomplete data, fetal anomalies or demise, history of cesarean delivery, spontaneous labor, and multiple gestation were excluded from the data set (n=4594) (Figure 1). TOI was defined as the time of the initial dose of cervical ripening agent or synthetic oxytocin, whichever occurred earlier, with subsequent analyses of induction methods. IOL duration was calculated by subtracting TOI from the time of delivery. Differences in TOI were analyzed for the remaining participants undergoing induction at term (n=3363). The Table summarizes the demographic and clinical characteristics of the study population. Body mass index (BMI) categories were defined according to the Centers for Disease Control and Prevention,^17^ with BMI <18.4 classified as underweight, 18.5 to 24.9 as normal weight, 25 to 29.9 as overweight, and >30 as obese. There were no underweight participants. Neonatal intensive care unit (NICU) admission included neonates admitted for NICU observation at Sparrow Hospital. Nulliparity was defined as parity 0 (P0) at the time of admission, whereas parous status was defined as parity ≥1 (P1+).

### Statistical analysis and visualizations

Data carpentry and visualizations were performed in the R environment (version 4.0.5; R Foundation for Statistical Computing, Vienna, Austria) using the *readxl* (version 1.3.1), *ggplot2* (version 3.3.3), *RColorBrewer* (version 1.1.2), *dplyr* (version 1.0.6), and *tidyr* (version 1.1.3) packages. Statistical tests, including analysis of variance (ANOVA), Kruskal–Wallis, and *t* tests, were performed using *rstatix* (version 0.7.0). Principal component analysis (PCA) was performed using the *factoextra* (version 1.0.7) and *FactoMineR* (version 2.4) packages. IOL duration was categorized as <12 hours, 12 to 24 hours, and >24 hours. Time-to-event analysis of total IOL duration was performed using a standard survival analysis and Cox proportional hazards analysis with the *survival* (version 3.2.11) and *survminer* (version 0.4.9) packages. Delivery was considered the event, and hazard ratios (HRs) were reported with 95% confidence intervals (CIs) as HR (95% CI). All data were used as the reference for comparisons between each bin and the rest of the sample. The rhythmic component of TOI was identified using the Lomb–Scargle periodogram for circadian rhythm analysis.^18,19^ This method is particularly well-suited for our data set given that labor and delivery data consist of nonuniform, independent measurements rather than continuous, frequent measurements from the same sample. The analysis was performed by separating TOI into 1-hour bins, yielding a total of 24 periods, and plotting the IOL duration for each period. The significance level of the periodogram was set at *α*=0.01, with the null hypothesis that IOL duration was not periodic. The peak periods were identified, representing the TOI associated with the greatest variation in the data set. Normalized power was used as a goodness-of-fit measure to quantify the strength of the rhythm. To retain statistical power for downstream analyses, data were further stratified into 3-hour bins, with differentiation by IOL agent type or delivery type, unless otherwise stated in the text.

## Results

### Baseline population characteristics

We first compared demographic and clinical variables of participants induced from 7 am to 7 pm (day) and from 7 pm to 7 am (night) (Table 1). We did not detect differences in maternal age (*P*=.653), ethnicity (*P*=.822), BMI groups (*P*=.532), parity (*P*=.135), gestational age (*P*=.478), or delivery method (*P*=.306). When induction groups were further split in 3-hours bins, statistically significant differences were found only in parity (*P*=.034) (Supplemental Table S1). Hence, the effect of parity as a covariate was considered in subsequent analyses.

In line with previous studies,^20^ we observed that the duration of IOL was significantly longer among participants who had a cesarean delivery than those who had a vaginal delivery (25.92 and 22.12 hours, respectively, in P0 population; *P*<.001, Welch 2-sample *t* test; 19.01 and 13.70 hours, respectively, in P1+ population; *P*<.001, Welch 2-sample *t* test). Parous participants with IOL duration <12 hours had a significantly reduced odds ratio (OR) of cesarean delivery (OR [95% CI], 0.370 [0.2862–0.514]; *P*<.001) compared with induction cohorts with longer IOL duration. Participants with IOL duration >24 hours had a significantly increased OR of cesarean delivery, including both nulliparous (OR [95% CI], 1.565 [1.278–1.917]; *P*<.001) and parous participants (OR [95% CI], 3.290 [2.286–4.684]; *P*<.001). Nulliparous participants had a significantly reduced OR of cesarean delivery with IOL duration between 12 and 24 hours (OR [95% CI], 0.663 [0.540–0.814]; *P*<.001) (Supplemental Figure S1).

### Induction of labor duration is shortest with induction during early morning hours

**FIGURE 2:**
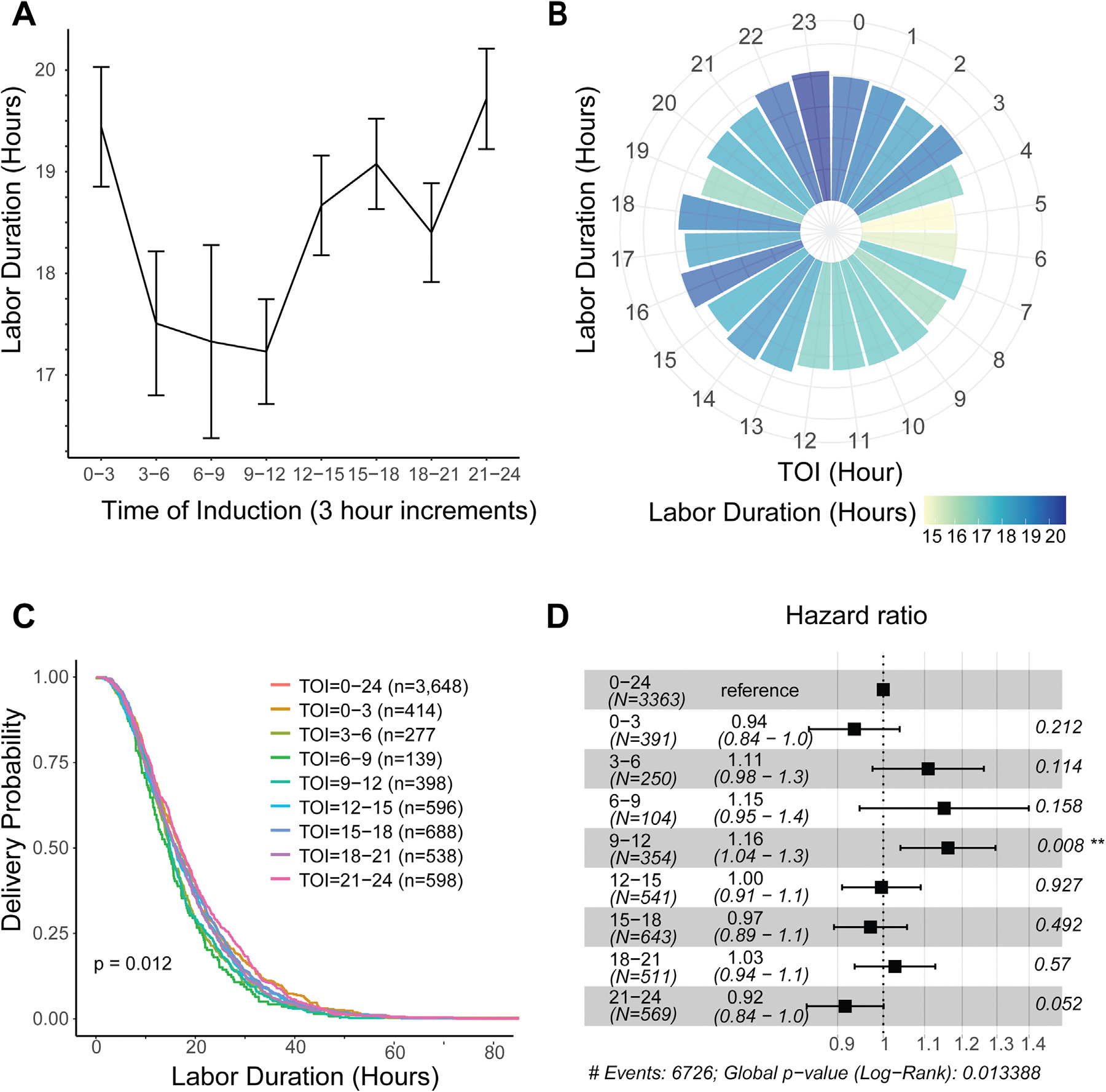
TOI defines labor duration in term participants **A,** The average labor duration for TOI in 3-hour increments shows significant differences (*P*<.001, 1-way analysis of variance). **B,** Splitting TOI into 1-hour bins is shown as a clock-like pattern from midnight to 11:00 pm. IOL duration differed significantly between TOI bins (*P*=.001, Kruskal–Wallis): durations are indicated by a color gradient ranging from shorter durations in yellow, through green, to longer durations in blue. Post hoc analysis using the Dunn test can be found in Supplemental Table S2. Time-to-event analysis was used to identify differences in IOL duration across TOI 3-hour bins and applied using **C,** Kaplan–Meier curves and **D,** Cox proportional hazards analysis. Delivery probability was defined as the probability of giving birth after induction initiated during a given time bin. The event for the hazard ratio (HR) was defined as giving birth. Higher HR values denote a higher probability of reaching birth in that TOI bin compared with any other time of day (midnight to 11:00 pm) as a reference. Significant differences, *P* values, and n/bin are indicated in each panel (**P<.01).

Given the known influence of circadian rhythms on physiological processes and birth,^10^ we examined whether the TOI impacted labor progression and total IOL duration. We observed significant differences in IOL duration (*P*=.012, 1-way ANOVA) (Figure 2, A) when data were divided into 3-hour intervals from midnight to midnight. Participants induced from 9:00 am to noon (adjusted *P*=.02) had significantly shorter labor than participants induced from 9:00 pm to midnight. Inducing labor from 9:00 am to noon resulted in an average IOL duration of 17.2 hours, compared with 19.7 hours observed at the longest duration with induction between 9:00 pm and midnight. Periodogram analysis using the Lomb–Scargle method revealed that IOL duration was rhythmically dependent on TOI (*P*=.005) (Supplemental Figure S2, A). Moreover, by further separating the data into 1-hour bins, we observed that initiating induction in the early morning resulted in shorter IOL durations compared with other times of the day, with the shortest average induction at 5:00 am (average of 14.8 hours) (Figure 2, B). IOL duration gradually increased throughout the day, peaking with TOI at 11:00 pm, resulting in an average IOL duration of 21.0 hours. Total IOL duration differed significantly across TOIs (*P*=.004, Kruskal–Wallis test). Results of pairwise post hoc analysis using the Dunn test are provided in Supplemental Table S2.

Next, we assessed the impact of TOI on IOL duration by induction agent (Supplemental Figure S3) and on labor progression (Supplemental Figure S4). TOI had a significant impact among participants who were initially induced with only synthetic oxytocin (*P*=.033, Kruskal–Wallis) or Cytotec (Pfizer) as the cervical ripening agent (*P*=.008), but not among those who underwent mechanical induction with a Foley catheter (*P*=.297) or combined Cytotec and synthetic oxytocin induction (*P*=.146) (Supplemental Figure S3). Moreover, we detected TOI-associated differences in duration from initial IOL to 6-cm cervical dilatation (*P*=.05, Kruskal–Wallis), but not from 6-cm cervical dilatation to the second stage of labor (*P*=.58) or from the second stage of labor to delivery (*P*=.43) (Supplemental Figure S4), suggesting that TOI preferentially affects cervical ripening and latent labor.

### Time of day of labor induction influences delivery probability and type without impacting infant outcomes

**FIGURE 3:**
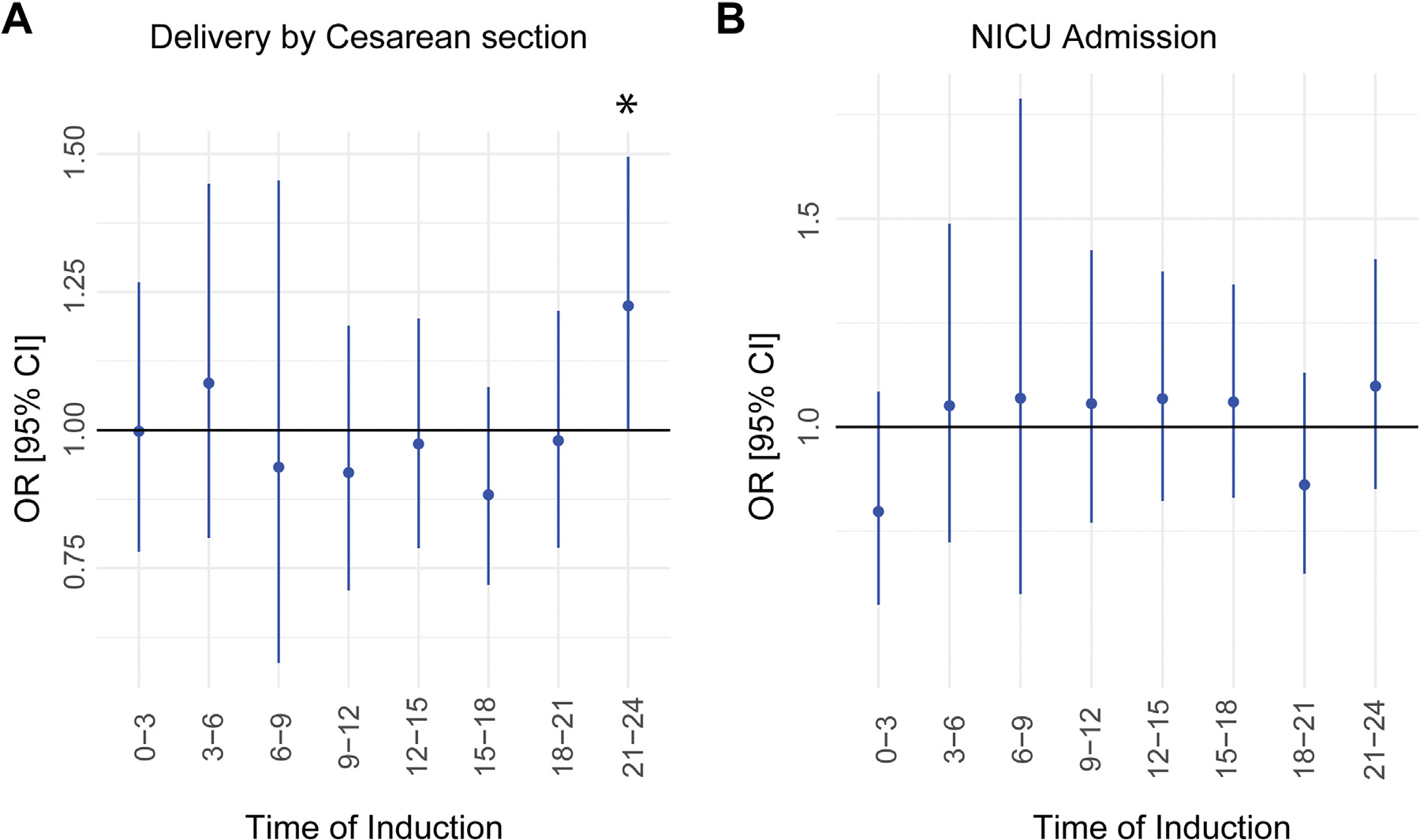
TOI defines risk of cesarean section, but not NICU admission **A,** OR of cesarean delivery (n=853 total) is significantly increased with induction from 9:00 pm to midnight (*P*=.048; n=569) compared with induction at any other time point. **B,** OR of NICU admission (n=493 total) did not significantly differ across TOI bins (Supplemental Table S3 includes individual *P* values). In each panel, the point denotes OR, with the 95% CI indicated by blue lines. OR=1 is indicated by the horizontal black line.

Standard time-to-event analysis identified significant differences in the probability of vaginal delivery based on TOI (*P*=.012) (Figure 2, C), with increased odds of vaginal birth with induction between 9:00 am and noon (OR [95% CI], 1.16 [1.04–1.3]; *P*=.008) (Figure 2, D). To further determine whether TOI would define the labor outcome, we examined the relationship between TOI and delivery methods (ie, vaginal and cesarean delivery), NICU admission, and resulting infant outcomes (Figure 3). We found that the probability of having a cesarean delivery was significantly increased with induction initiation from 9:00 pm to midnight compared with induction at any other time points (OR [95% CI], 1.22 [1.00–1.50]; *P*=.048) (Figure 3, A; Supplemental Table S3). Importantly, no significant differences in the probability of NICU admission across all 3-hour TOI bins were identified (Figure 3, B). The list of *P* values and ORs for each TOI bin is provided in Supplemental Table S3. Likewise, we found no significant difference in infant diagnoses based on TOI for sepsis (*P*=.41), respiratory distress (*P*=.55), transient tachypnea (*P*=.59), hypoglycemia (*P*=.22), feeding difficulties (*P*=.30), or jaundice (*P*=.66).

### Maternal factors condition the effect of time of day of labor induction on labor duration

**FIGURE 4:**
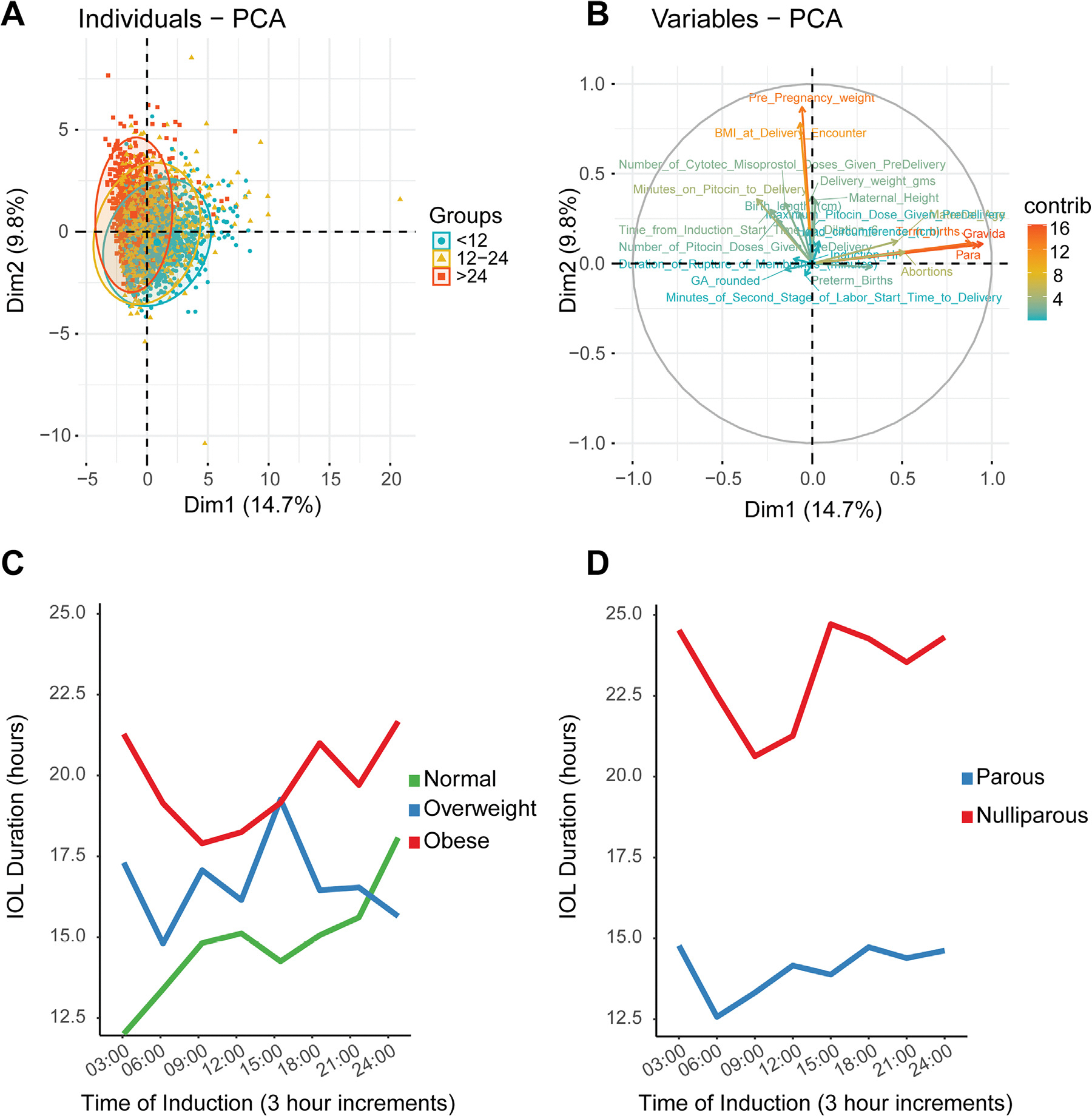
Multivariate analysis revealed clusters according to IOL duration **A,** PCA was performed, and the plot of individuals was grouped by IOL duration >24 hours (red), 12 to 24 hours (yellow), and <12 hours (blue). **B,** PCA graphic of variables plotted with the direction and contribution for each variable in the PCA. Positively and negatively correlated variables point to the same or opposite side of the plot, respectively. Contribution strength is indicated by arrow length and color gradient, with longer arrows and red color indicating higher contribution, and shorter length and blue color indicating lower contribution. Multivariate effect of TOI on labor duration with respect to top contributing variables: **C,** maternal BMI and **D,** parity. BMI group (*P*=.05, 2-way analysis of variance [ANOVA] controlling for delivery method) is indicated by color for normal weight (green) (n=183), overweight (blue) (n=869), and obese participants (red) (n=2311). Parity (*P*=.26, 2-way ANOVA controlling for delivery method) is indicated by color, with nulliparous participants (n=1576) defined as parity 0 (P0) and red in color, whereas parous participants (n=1787) are defined as parity ≥1 (P1+) and indicated by blue color.

To identify patterns and key contributors to variation in IOL duration, we performed PCA incorporating maternal and labor-related variables. According to 2-dimensional plotting (Figure 4, A), there was continuous separation, with participants whose IOL duration exceeded 24 hours (red dots) distinct from those who labored 12 to 24 hours (yellow dots) or <12 hours (blue dots). Analysis of variables (Figure 4, B) revealed prepregnancy weight and BMI as the top variables contributing to this separation in dimension 2 (Dim2, y-axis). Other top contributors in Dim2 include labor induction variables, such as the number of doses, minutes from induction to delivery, and time from induction to dilatation of 6 cm. Top contributors in dimension 1 (Dim1, x-axis) include maternal variables such as gravidity, parity, and number of term births. We further stratified the data on the basis of the key variables identified by PCA and analyzed the interaction effects of TOI with maternal BMI and parity on IOL duration:

1. Maternal BMI (Figure 4, C): To assess how BMI impacted outcomes, we conducted a 2-way ANOVA, which revealed that both TOI (F[7, 3339]; *P*=.010) and maternal BMI (F[2, 3339]; *P*<.001) have statistically significant effects on IOL duration. However, there was no statistically significant interaction between these effects (F[14, 3339]; *P*=.080). Participants with a normal-range BMI experienced the shortest labor duration when induced from midnight to 3:00 am (12.06±6.50 hours; n=19), with the longest IOL duration observed for induction between 9:00 pm and midnight (17.81±9.63 hours; n=30) (green line). However, when testing for circadian rhythmicity, normal-weight participants did not exhibit a significant circadian rhythm in IOL duration as a function of TOI (*P*=.47; n=183) (Supplemental Figure S2, B). Participants with obesity experienced the shortest interval to delivery with TOI between 6:00 am and 9:00 am (17.64±9.38 hours; n=73), with peak durations from 3:00 pm to 6:00 pm (20.59±11.82 hours; n=433) and again from 9:00 pm to midnight (21.22±12.06 hours; n=407) (red line). Circadian rhythmicity was statistically significant in the obese population (*P*=.003; n=2311) (Supplemental Figure S2, D).
2. Parity (Figure 4, D): Next, we assessed whether parity impacted IOL duration. Two-way ANOVA revealed that both TOI (F[7, 3347]; *P*=.003) and parity (F[1, 3347]; *P*<.001) have statistically significant effects on IOL duration, but there was no statistically significant interaction between these effects (F[7, 3347]; *P*=.271). Consistent with the literature,^21^ mean IOL duration across the whole day was longer in nulliparous participants than in parous participants (24.90±11.82 and 14.42±8.81 hours for nulliparous and parous participants, respectively; *P*<.001, Welch 2-sample *t* test). Of note, the mean IOL duration was shortest with induction during morning hours regardless of parity. Nulliparous participants experienced the shortest IOL duration when induced between 6:00 am and 9:00 am (20.63±10.31 hours), whereas parous participants experienced the shortest IOL duration when induced between 3:00 am and 6:00 am (12.57±6.76). Statistical testing did not detect significant circadian rhythmicity for nulliparous (*P*=.74; n=1576) or parous (*P*=.67; n=1787) participants.

**FIGURE 5:**
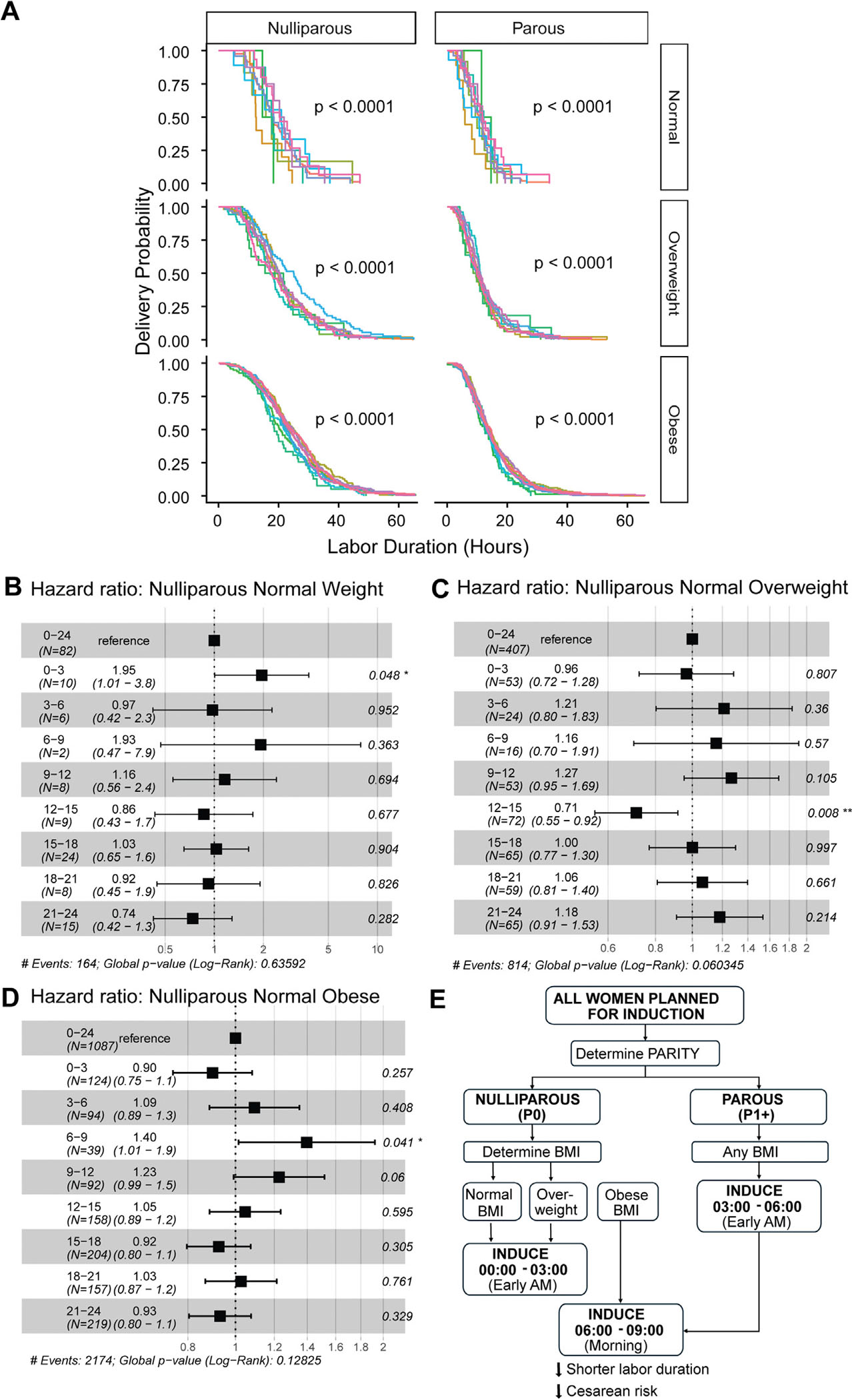
TOI modulates delivery probability as a function of BMI and parity Time-to-event analysis of IOL duration across TOI groups in 3-hour bins was stratified by normal weight (n=183), overweight (n=869), and obese (red) BMI groups across nulliparous (P0) and parous (P1+) participants. **A,** Kaplan–Meier curves were produced for each combination of BMI and parity and the resulting *P* values indicated in the plots. **B–D,** Delivery probability was defined as the probability of giving birth after induction initiated during the given time bin. Nulliparous overweight and nulliparous obese groups with significant differences in delivery probability across TOI were selected for Cox proportional hazards analysis. The event for the hazard ratio (HR) was defined as giving birth. Higher HR values denote a higher probability of reaching birth in that TOI bin compared with any other time of day (midnight to 11:00 pm) as a reference. Significance was set at α=0.05 with *P* values and n/bin indicated in each panel. **E,** Summary flowchart highlighting the optimal TOI to reduce labor duration and risk of cesarean delivery for each subgroup of BMI and parity.

Given the influence of maternal BMI and parity on labor dynamics, we further assessed how these factors modify the relationship between TOI and delivery probability by stratifying the time-to-event analyses (Figure 5). We found significant differences in delivery probability based on TOI across all BMI and parity subgroups, including nulliparous normal BMI, nulliparous overweight, nulliparous obese, parous normal BMI, parous overweight, and parous obese participants (all *P*<.001). Nulliparous participants with normal BMI had a significantly increased probability of delivery with induction between midnight and 3:00 am (HR [95% CI], 1.95 [1.01–3.80]; *P*=.048), whereas nulliparous participants with obesity had increased odds of delivering with induction from 6:00 am to 9:00 am (HR [95% CI], 1.40 [1.01–1.90]; *P*=.041). In contrast, nulliparous overweight participants had significantly decreased odds of delivering with induction between noon and 3:00 pm (HR [95% CI], 0.71 [0.55–0.92]; *P*=.008). Parous participants did not exhibit significant differences by TOI.

### Comment

#### Principal findings

Herein, we describe that the time of day of IOL is associated with a circadian rhythm in IOL duration. Differences are specifically identified in the first stage of labor from initial induction to cervical dilatation of 6 cm. Importantly, we show that the optimal TOI is conditioned by BMI and parity, and that considering TOI may be most beneficial for nulliparous and/or obese participants (Figure 5, E).

#### Results in the context of what is known

Comparative analysis of TOI demonstrated differences in IOL duration shaped by known maternal factors^12,22–24^ and highlighted the consistent impact of maternal factors on induction success and delivery outcomes. Importantly, we confirm that BMI and parity are significant contributing factors to labor duration.^25^ Stratifying by BMI and parity revealed that obese and nulliparous participants were most susceptible to experiencing a longer labor duration following IOL. Nulliparous participants with normal, overweight, and obese BMI differed significantly in their IOL durations across TOI groups, demonstrating that nulliparous obese women stand to benefit the most from scheduling induction from 3:00 am to 9:00 am. Indeed, the relationship between circadian rhythm, obesity, and diabetes has been well-established,^14,26,27^ and an increased risk of obesity is associated with circadian dysfunction caused by shift work.^28,29^ Furthermore, our recent work in a smaller cohort identified differences in IOL duration by time of day of induction among participants with gestational diabetes mellitus.^12^ Importantly, this previous study^12^ showed that synchronized human myometrial cells exhibited time-of-day differences in their contractile response to oxytocin, supporting the hypothesis that time-of-day differences in oxytocin IOL duration might be driven by the myometrium’s contractile response to oxytocin. Furthermore, the study used transgenic mice to demonstrate that loss of the core circadian clock gene *Bmal1* in the uterine smooth muscle resulted in downregulation of the oxytocin receptor and reduced uterotonic efficacy of oxytocin. Future work will aim to determine whether the benefit of morning IOL in reducing labor duration is driven by daily changes in oxytocin receptor expression or signaling in the myometrium.

#### Clinical and research implications

Understanding the factors that influence IOL duration is critical for improving maternal outcomes and optimizing resources used in obstetrical care. Our identification of TOI as a key determinant of IOL duration introduces a biologically informed and cost-effective strategy to enhance labor management. Unlike prior studies that have focused primarily on hospital staffing and shift structures,^30–32^ our findings identify that maternal circadian biology modulates cervical ripening and latent labor progression. Although previous analyses treating TOI as a binary variable found no effect on IOL duration,^33^ our more granular approach reveals significant differences, particularly in cervical ripening and latent labor. No differences were identified across TOI for the remaining stages of labor up to delivery. The significant shortening of labor from initial induction to cervical ripening and latent labor is likely related to oxytocin receptor effects beyond myometrial contractility. Specifically, oxytocin receptors are found in the decidua and chorioamniotic membranes, where they play a role in prostaglandin and inflammatory mediator production, which are critical to cervical ripening and early labor.^34^ In other words, our findings indicate that the differences in total IOL duration across TOI groups are likely due to the diverse actions of the oxytocin receptor late in pregnancy and early parturition rather than the availability of hospital resources, highlighting the mechanistic role of circadian rhythms in labor onset during the late evening and early morning hours.^35^ These results underscore the importance of considering both biological timing and clinical logistics when designing induction protocols and staffing models.

Importantly, differences in TOI across maternal characteristics were not associated with adverse neonatal outcomes. This finding is supported by other studies that found no differences in maternal or neonatal outcomes between morning and evening induction.^36^ Importantly, we identified an increased rate of cesarean delivery with IOL initiation from 9:00 pm to midnight, coinciding with the longest IOL duration among TOI groups. Hence, our study found that initiating induction at an optimal time of day based on participant characteristics has the potential to reduce IOL duration and mitigate the risk of cesarean delivery without increasing NICU admission or adverse infant diagnoses. This indicates that considering TOI in the management of induced participants does not result in negative maternal or fetal outcomes.

#### Strengths and limitations

The current analysis aimed to evaluate the general impact of TOI on IOL duration while accounting for maternal characteristics. Additional analyses should be performed to study vaginal deliveries specifically, as well as differences across IOL methods and dosage of synthetic oxytocin. Such analyses were not feasible because of the study size and data availability; however, our previous study found a comparable reduction in IOL duration with midnight induction among participants with gestational diabetes, independent of delivery method.^12^

TOI may also influence other out come measures such as maternal and provider satisfaction, workflow efficiency, and health care resource utilization. Such measures were not collected in our data sets, and further studies focused on quality of care are warranted.

It is worth noting that TOI may be influenced by clinical (eg, cervical readiness at the time of induction, Bishop score) and personal (eg, request for the presence of a partner during delivery) factors. Because our analysis was limited to participants who underwent induction, our findings might reflect selection bias relative to the general population. To mitigate the possible effects of confounding factors, we performed multi-variable and survival model analyses. Multivariable analysis enabled adjustment for other variables that could influence the outcome (eg, age, parity, BMI, and Bishop score) and therefore the discrimination of the effect of TOI from those variables. Likewise, the use of survival models for IOL duration analysis accounted for the inherent variability in labor length independent of the time of induction. It should also be noted that the normal BMI group was underpowered, and these data should be interpreted with caution.

Despite the availability of guidelines for labor and delivery, variations in logistics (eg, staffing models) and clinical practice (eg, protocols) across institutions may influence the impact of TOL on IOL duration. In the present study, we used data from a single institution to mitigate such variation and focus on the effects of the circadian rhythm. Specifically, the institution our data were retrieved from did not have a policy restricting IOL timing on the basis of indication or Bishop score during the study period. Validation of these findings in a multicentric study accounting for interinstitutional logistic variables will be necessary before implementing our results in clinical practice.

Scheduling labor induction in the morning may be impractical for specific institutions because of staffing constraints. To address this, our analysis provides stratification to identify maternal groups that would most benefit from scheduling induction during a specific time frame. On the basis of the data presented, we propose that every effort should be made to provide induction during the optimal time for at-risk populations according to staff and resource availability (Figure 5, E).

## Conclusions

This study provides strong evidence for the use of TOI in labor management. Nulliparous participants, who are most prone to increased labor duration, could benefit the most from initiating elective induction in the early morning between 3:00 am and 9:00 am, a time window that is associated with shorter IOL duration across all groups. ■

## Supplementary Material

1

2

3

Supplementary material associated with this article can be found, in the online version at doi:10.1016/j.ajogmf.2026.101898.

## Figures and Tables

**TABLE:** Population statistics of study participants comparing induction of labor initiation during the day (7 am to 7 pm) and night (7 pm to 7 am)

Characteristics	Day (n=1756)	Night (n=1607)	All (N=3363)	*P* value
Maternal age (y)				
Mean (SD)	28.8 (5.37)	28.8 (5.52)	28.8 (5.44)	.653^a^
Median (min, max)	29.0 (18.0, 47.0)	29.0 (18.0, 47.0)	29.0 (18.0, 47.0)	
Race				
White	1223 (69.6%)	1073 (66.8%)	2296 (68.3%)	.822^b^
Black	252 (14.4%)	245 (15.2%)	497 (14.8%)	
Hispanic	91 (5.2%)	84 (5.2%)	175 (5.2%)	
Asian	57 (3.2%)	66 (4.1%)	123 (3.7%)	
Other	133 (7.6%)	139 (8.6%)	272 (8.1%)	
BMI				
Normal	101 (5.8%)	82 (5.1%)	183 (5.4%)	.532^b^
Overweight	472 (26.9%)	397 (24.7%)	869 (25.8%)	
Obese	1183 (67.4%)	1128 (70.2%)	2311 (68.7%)	
Parity				
Nulliparous	794 (45.2%)	782 (48.7%)	1576 (46.9%)	.135^b^
Parous	962 (54.8%)	825 (51.3%)	1787 (53.1%)	
Gestational age (wk)				
Mean (SD)	39.4 (1.25)	39.3 (1.19)	39.4 (1.22)	.478^a^
Median [min, max]	39.4 (37.0, 41.9)	39.3 (37.0, 41.9)	39.3 (37.0, 41.9)	
Delivery method				
Cesarean delivery	426 (24.3%)	427 (26.6%)	853 (25.4%)	.306^b^
Vaginal	1330 (75.7%)	1180 (73.4%)	2510 (74.6%)	

*BMI*, body mass index.

a One-way analysis of variance;

b Chi-squared test.

## AJOG MFM at a Glance

### Why was this study conducted?

Although spontaneous labor follows clear circadian patterns, it is not well understood whether the time of day of initiation of medically induced labor influences its duration. This study was undertaken to evaluate whether the time of day when labor induction begins affects the duration of induction of labor (IOL), and to explore how maternal factors such as body mass index and parity may shape this effect.

### Key findings

IOL duration follows a circadian rhythm and is significantly shorter when induction is initiated between 3:00 am and 9:00 am. The difference is most pronounced in the first stage of labor, from induction to cervical dilatation of 6 cm. Nulliparous and obese participants benefit the most from morning inductions. Time of day of induction can reduce cesarean delivery rates without affecting neonatal intensive care unit admissions or adverse neonatal outcomes.

### What does this add to what is known?

This large-scale retrospective study demonstrated that the time of day of initiating IOL impacts IOL duration in term pregnancies. This study introduces a biologically informed and cost-neutral approach to optimize labor management, especially for higher-risk groups such as obese or nulliparous participants, while also reducing cesarean delivery rates.
