# Supplementary material for: Time of day of induction impacts the total duration of labor
Source: Am J Obstet Gynecol MFM. Author manuscript; Available in PMC 2026 Jul 17. (PMC13377848; doi:10.1016/j.ajogmf.2026.101898)
Supplement: 1 [file NIHMS2189476-supplement-1.docx]

**Supplementary Material**

**Time of Day of Induction Impacts the Total Duration of Labor**

Kylie Cataldo^1^, Robert Long^3,4,5^, Isoken Olomnu^3^, Rene Cortese^1,2#*^, and Hanne M. Hoffmann^6^

**Supplementary Figure S1**

**
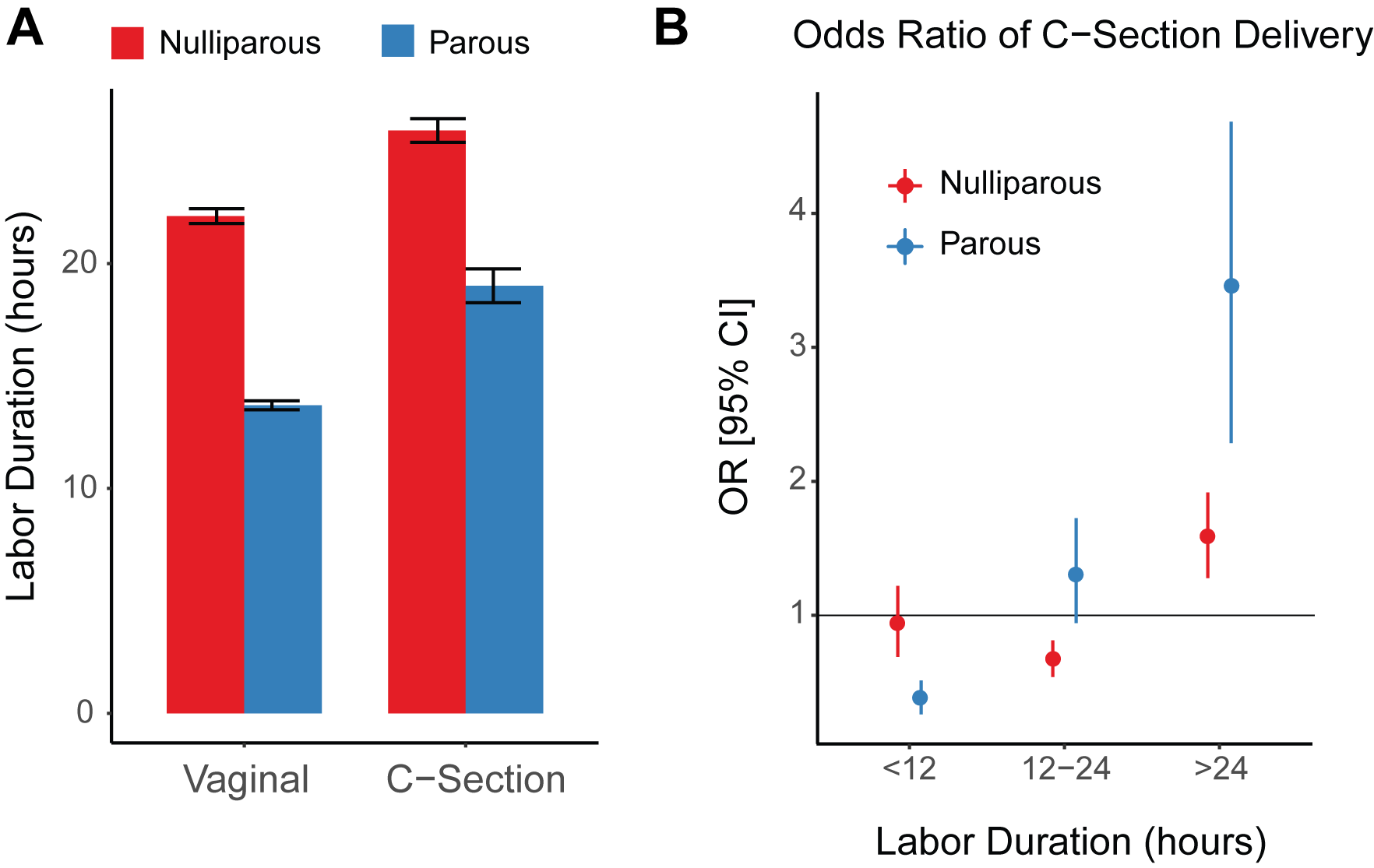
**

**Supplementary Figure 1. Longer labor duration increases the odds of delivering by cesarean section. A**) Average labor duration for vaginal (n=2,510) and cesarean (n=853) deliveries for nulliparous (n=1,576) and parous (n=1,787) subjects. Labor duration is longer for nulliparous subjects and deliveries resulting in cesarean section (p<0.001, Welch’s two-sample T-test).  **B**) Odds ratio (OR) of delivering by cesarean section across labor duration bins for nulliparous and parous subjects. The point in panel B denotes OR with the 95% confidence interval (CI) indicated by the colored lines. OR=1 is shown with the horizontal black line. OR for cesarean section in parous subjects with labor duration less than 12 hours was significantly lower than in all other groups (OR [95% CI] = (0.370 [0.262, 0.514], p<0.001). In deliveries with labor duration greater than 24 hours, OR for cesarean section was significantly higher compared with the other groups for both, nulliparous (1.565 [1.278, 1.917], p<0.001) and parous subjects (3.290 [2.286, 4.684], p<0.001). Subjects in the nulliparous group with labor duration between 12 and 26 hours had reduced OR for cesarean section (0.663 [0.540, 0.814], p<0.001).

**Supplementary Figure S2**


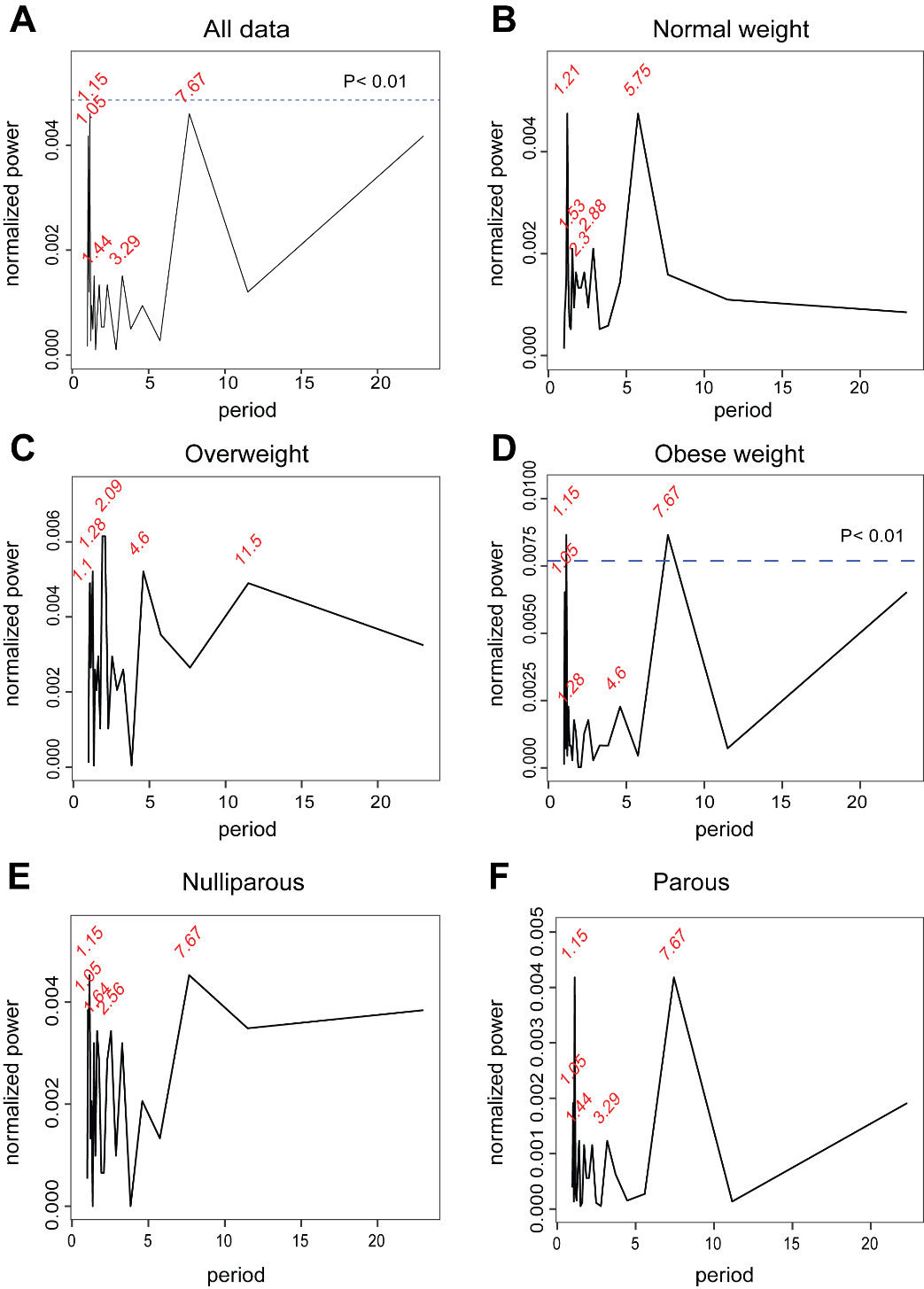


**Supplementary Figure S2. TOI impacts labor duration in a circadian manner and is influenced by BMI**. The circadian rhythm analysis of TOI is shown using Lomb-Scargle Periodograms. The analysis was performed by separating TOI in one-hour bins for a total of 23 periods (x-axis) and α = 0.01 for identifying statistical significance, indicated by the dashed horizontal line. Specific peaks are identified in red for TOI bins responsible for the greatest variation in the data. Normalized power measures strength of the rhythm. **A)** There was a significant circadian rhythm of induction of labor (IOL) duration across the entire dataset as a result of induction time, identified by significant peaks at 1.15 hours (p=0.005, n=3,363). Separating by BMI reveals no significant peaks in **B)** normal weight (p=0.47, n=183), **C)** Overweight (p=0.94, n=869) pregnancies, whereas **D)** obese subjects have significant differences in circadian rhythm (p=0.003, n=2,311). Separating by parity did not identify significant peaks in either the **E)** nulliparous (p=0.74, n=1,576) or parous (p=0.67, n=1,787) populations.

**Supplementary Figure S3**


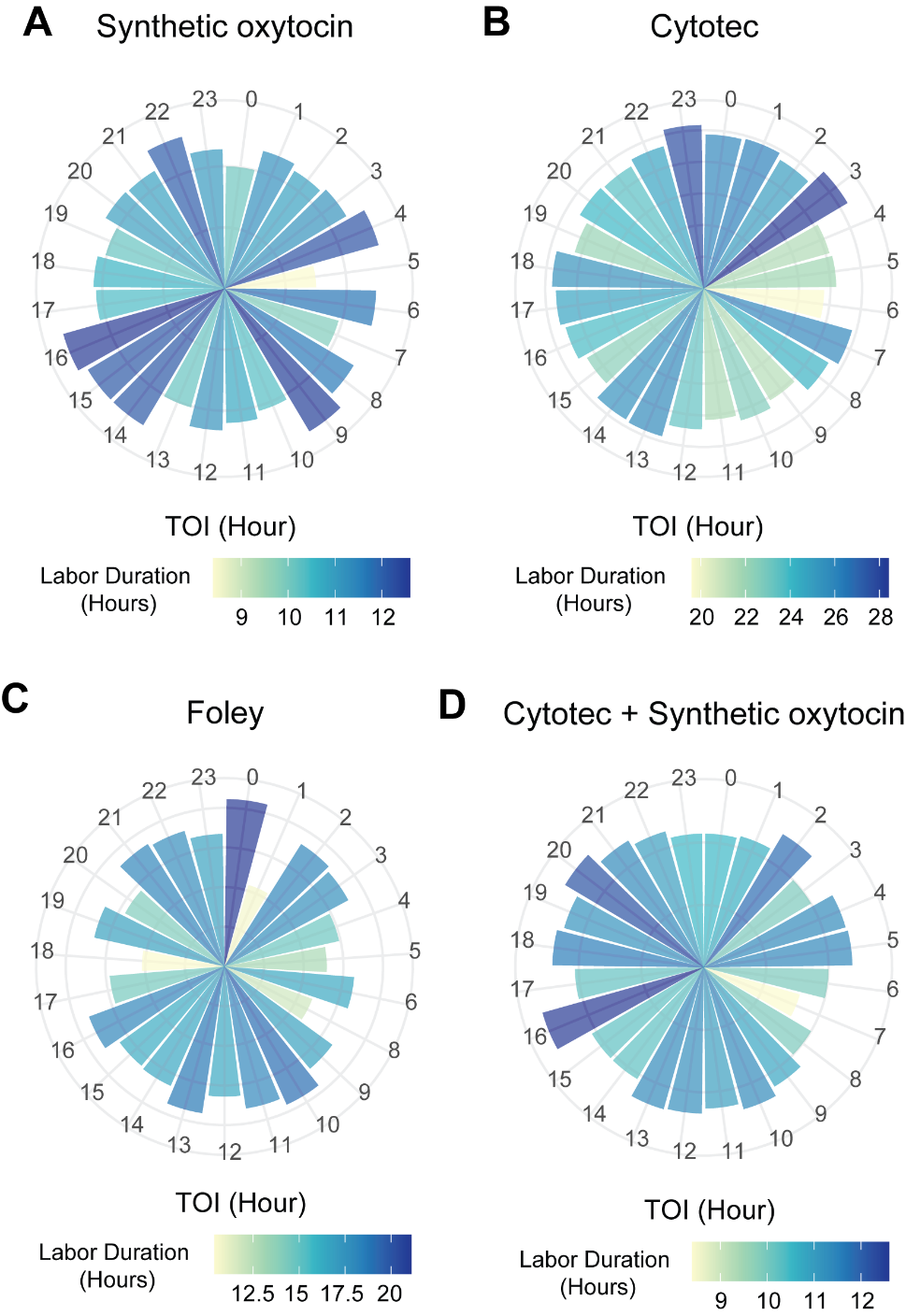


**Supplementary Figure 3. Induction of labor duration across induction times stratified by induction start method.** Average induction of labor (IOL) duration across 1-hour time of induction (TOI) bins is shown in a clock-like pattern from 00:00 hours (midnight) to 23:00 hours and stratified by induction start method using **(A)** synthetic oxytocin (p=0.033, Kruskal-Wallis), **(B)** Cytotec (p=0.008), **(C)** mechanical induction with foley catheter (p=0.297), and **(D)** synthetic oxytocin and Cytotec (p=0.146). IOL duration is indicated by color gradient from shorter durations in yellow, over green, to longer durations in blue.

**Supplementary Figure S4**


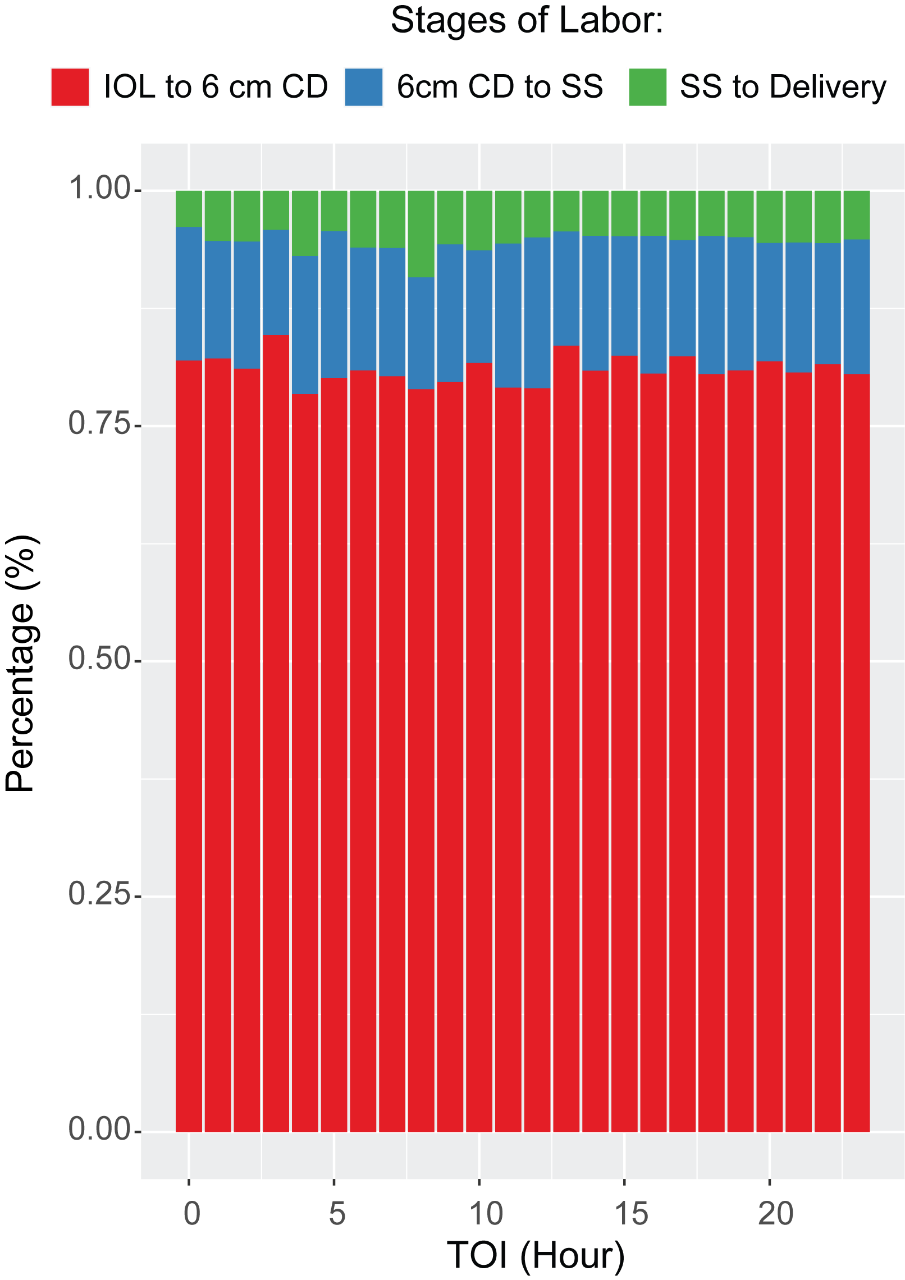


**Supplementary Figure S4. Labor progression across induction times**. Percentages of total time in each stage of labor across 1-hour time of induction (TOI) bins. Stages of labor were defined as time from induction of labor (IOL) to cervical dilation (CD) of 6 cm (red) (p=0.05, Kruskal-Wallis), time from cervical dilation of 6 cm to second stage (SS) of labor (blue) (p=0.57, Kruskal-Wallis), and time from second stage of labor to delivery (green) (p=0.43, Kruskal-Wallis).

**Supplementary Tables**

**Supplementary Table 1.** **Population statistics of study participants comparing induction of labor across 3-hour increments.**

(Provided as an excel sheet)

**Supplementary Table 2. Dunn Test Post-Hoc Analysis of time of induction (TOI) in 1-hour bins.***

(Provided as an Excel sheet)

**Supplementary Table 3. Odds ratios (OR) of delivery method and NICU admissions.**

| **Delivery method** | | | | |
| --- | --- | --- | --- | --- |
| TOI-3hr^1^ | n (c-section) | n (vaginal) | p-value^2^ | OR [95% CI] |
| 0-3 | 99 | 292 | 0.98 | 0.998 [0.780, 1.268] |
| 3-6 | 67 | 183 | 0.59 | 1.085 [0.806, 1.446] |
| 6-9 | 25 | 79 | 0.75 | 0.933 [0.579, 1.453] |
| 9-12 | 85 | 269 | 0.54 | 0.923 [0.710, 1.189] |
| 12-15 | 135 | 406 | 0.81 | 0.975 [0.786, 1.202] |
| 15-18 | 151 | 492 | 0.22 | 0.882 [0.720, 1.078] |
| 18-21 | 128 | 383 | 0.86 | 0.981 [0.787, 1.217] |
| 21-24 | 163 | 406 | 0.05 | 1.22 [1.000, 1.495] |
| **NICU admission** | | | | |
| TOI-3hr^1^ | n (admitted) | n (not admitted) | p-value^2^ | OR [95% CI] |
| 0-3 | 48 | 343 | 0.16 | 0.800 [0.573, 1.085] |
| 3-6 | 38 | 212 | 0.80 | 1.051 [0.723, 1.488] |
| 6-9 | 16 | 88 | 0.83 | 1.069 [0.598, 1.789] |
| 9-12 | 54 | 300 | 0.74 | 1.056 [0.770, 1.424] |
| 12-15 | 83 | 458 | 0.62 | 1.068 [0.822, 1.373] |
| 15-18 | 98 | 545 | 0.64 | 1.060 [0.830, 1.342] |
| 18-21 | 67 | 444 | 0.28 | 0.861 [0.648, 1.128] |
| 21-24 | 84 | 480 | 0.47 | 1.098 [0.851, 1.403] |

^1^Time of induction in 3-hour bins

^2^Statistical comparisons performed using chi-squared test
